# Supplementary material for: Deacetylation induced nuclear condensation of HP1γ promotes multiple myeloma drug resistance
Source: Nat Commun. 2023 Mar 9;14:1290. doi: 10.1038/s41467-023-37013-x (PMC9998874; doi:10.1038/s41467-023-37013-x)
Supplement: Supplementary file 1 — Supplementary Information [file 41467_2023_37013_MOESM1_ESM.pdf]

Supplementary Figures and Legends

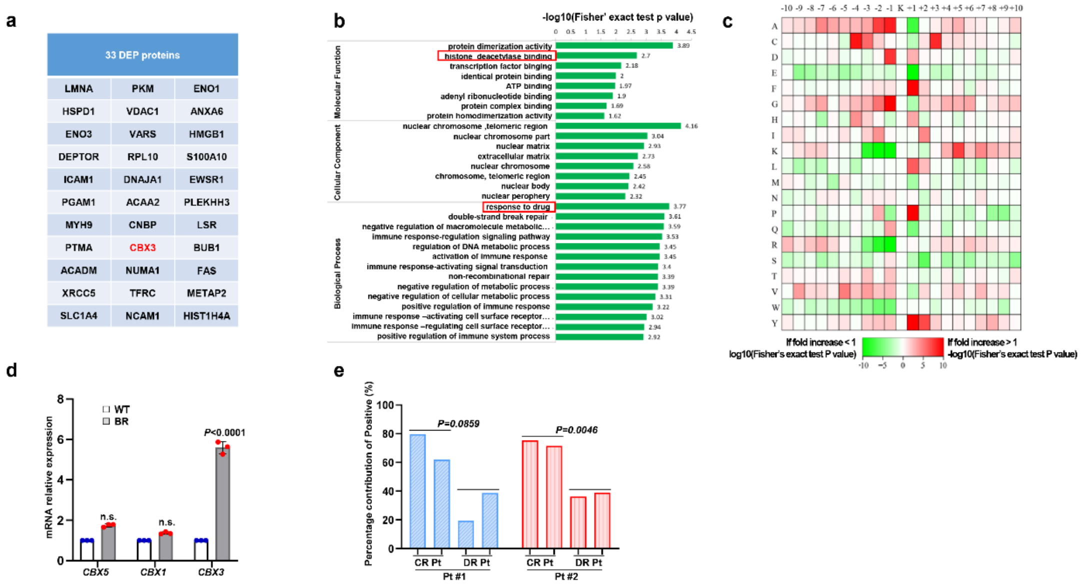

Supplementary Fig. 1: Deferentially expressed proteins in drug-resistant MM cells

a List of SILAC assay-identified 33 DEP proteins in proteomics and acetylome in BR and WT MM.1S cells. **b** GO enrichment analysis shows the biological processes and cellular functions of DEP proteins in the BR MM.1S cells. **c** Motif analysis of all identified acetylated sites in SILAC assay for the BR MM.1S cells. **d** QPCR detecting the genes of HP1 family genes in WT and BR MM.1S cells (mean ± s.d.; n = 3 independent experiments). **e** Quantification of the positive staining in Figure 1K, DR vs CR. *P* values were determined by Student's t test **(d, e)**. Source data are provided as a Source data file.

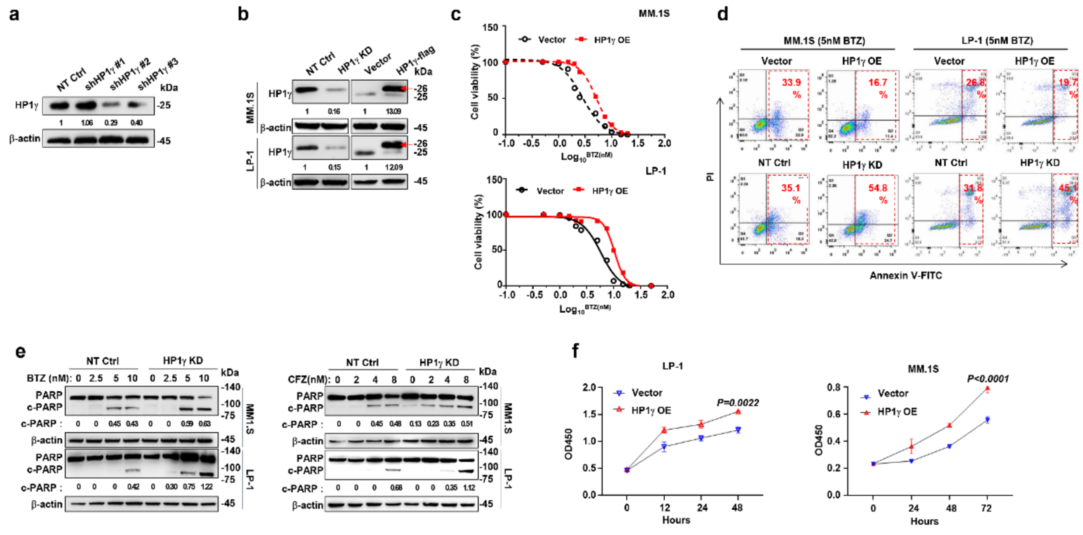

Supplementary Fig. 2: Effects of HP1γ on MM cell apoptosis. **a** Western blotting shows the efficacy of three shRNAs targeting HP1γ in HEK293T cells, and (**b**) validates the knockdown efficacy of shRNA#2 (HP1γ KD) and overexpression efficacy of HP1γ (HP1γ OE) in MM cells. **c** Alteration of IC50 to BTZ treatment in the Vector and HP1γ OE cells (n = 3). **d** Representative flow cytometry assay to detect the percentage of apoptotic cells in the Vector, HP1γ OE, non-target control (NT Ctrl) and HP1γ KD MM cells induced by 5 nM BTZ for 48 hr. **e** Cleavage of PARP as the apoptotic marker in MM.1S and LP-1 cells with HP1γ expression manipulated by shRNA or ectopic expression vector, treated with increasing dosage of BTZ and carfilzomib (CFZ) for 24 hr. **f** Cell proliferation of LP-1 and MM.1S cells stably expressing vector (Vector) or HP1γ OE (mean ± s.d.; n= 3 independent experiments). Differences between groups were assessed by one-way ANOVA test. Source data are provided as a Source data file.

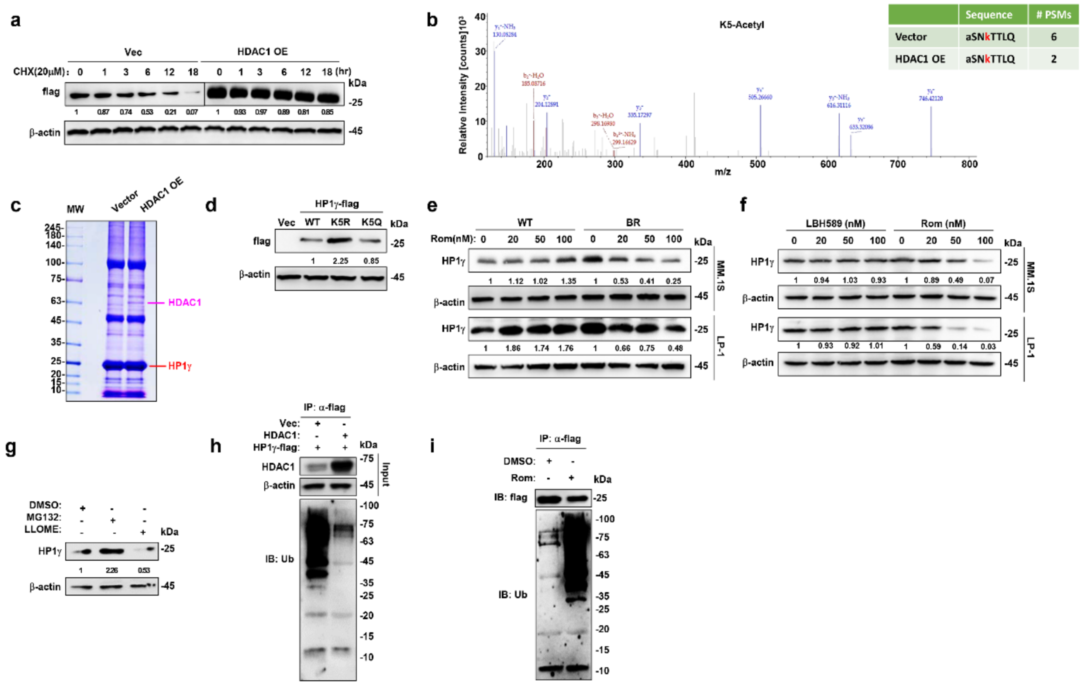

Supplementary Fig. 3: Acetylation modification enhances ubiquitination of HP1γ. **a** Degradation of HP1γ-flag protein in HEK293T transfected with HDAC1 and treated with 20 μM cycloheximide (CHX) for 12hr. **b** Mass spectra of enriched acetylated peptides containing K5 sites in HP1γ-flag proteins. Upper: Degree of enrichment of acetylated lysine sequences of HP1γ in Vector and HDAC1 OE groups. PSM (peptide-spectrum matches) is positively correlated with abundance. **c** Coomassie blue staining

for flag-pulldown in HEK293T cells transfected with vector or HP1γ-flag for 48 hr. **d** Western blotting shows the HP1γ protein level in the HEK293T cells transfected with flag-tagged HP1γ WT, K5R and K5Q. **e** Levels of HP1γ protein in WT and BR MM.1S and LP-1 cells treated with gradient concentration of Romidepsin (Rom) for 24 hr. **f** Western blotting shows the levels of HP1γ protein in MM.1S and LP-1 cells treated with gradient concentration of LBH589 and Rom for 24 hr. **g** Western blotting shows HP1γ levels in LP-1 cell treated with DMSO, MG132 (20μM) and LLOME (L-leucyl-L-leucine methyl ester, 20μM) for 24hr. Ubiquitination status of HP1γ in HEK293T cells transfected with **(h)** HDAC1 for 48 hr, or **(i)** Rom (100nM) for 24 hr. Source data are provided as a Source data file.

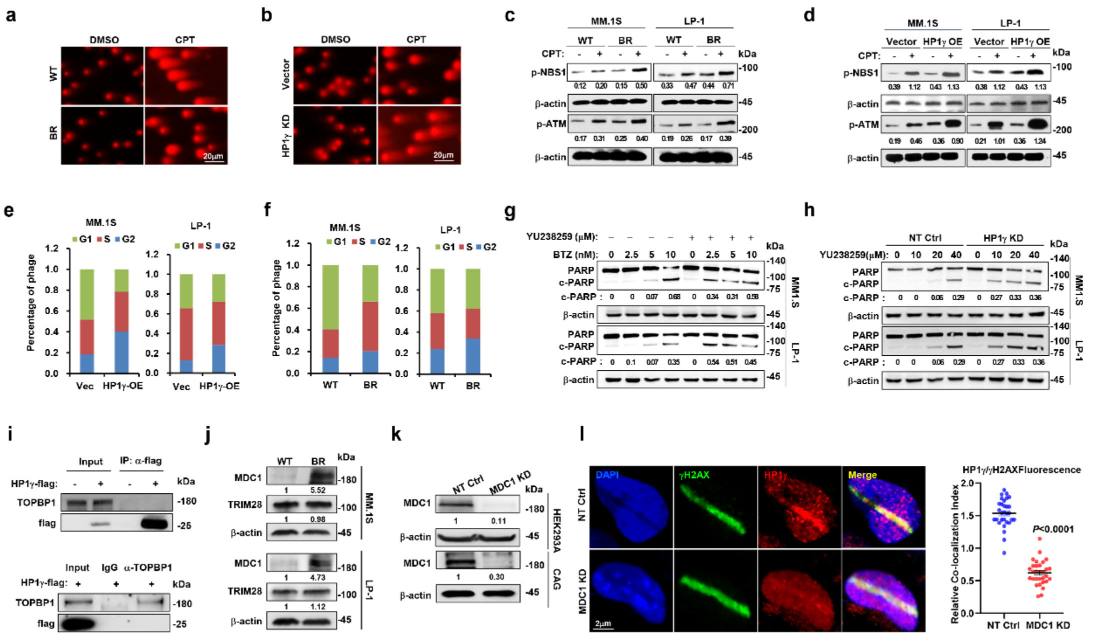

Supplementary Fig. 4: MDC1 recruits HP1γ and HP1γ facilitates homologous recombination. a Comet assay images showing DNA damage in WT and BR MM.1S cells treated with DMSO or CPT, and (**b**) in Vector and HP1γ OE MM.1S cells treated with DMSO or CPT. **c** Western blotting shows p-NBS1 and p-ATM levels in WT and BR, and in (**d**) Vector and HP1γ OE MM.1S and LP-1 cells. **e** Cell cycle assay of the WT, BR and **(f)** stably expressing Vector or HP1γ OE MM.1S and LP-1 cells. **g** Cleaved PARP in NT Ctrl and HP1γ KD cell treated with HR inhibitor-YU238259 for 24 hr, and (**h**) in MM cell treated with increasing dosage of BTZ combined with YU238259 for

24 hr. **i** Co-IP assay shows bilateral interactions between exogenous HP1γ with TOPBP1. **j** Levels of MDC1 and TRIM28 in the WT and BR MM.1S and LP-1 cells. **k** Knockdown efficacy of MDC1 KD in CAG and HEK293A cells. **l** Left: immunofluorescence assay demonstrating the HP1γ recruited to DNA damage regions in HEK293A cells. The focused regions of DNA damage were produced by using a scanning laser system. Right: the graph represents the relative co-localization index of the HP1γ protein on the γH2AX track. Error bars represent S.E.M., n= 3 independent experiments and differences relative to NT Ctrl were calculated using student's t test. Source data are provided as a Source data file.

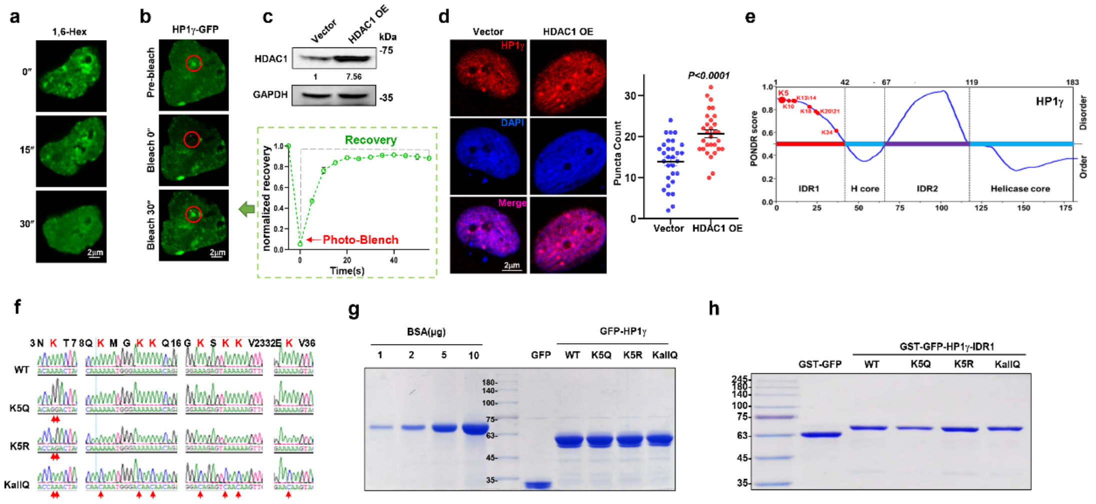

Supplementary Fig. 5: Deacetylation enhances nuclear condensation of HP1γ. a Fluorescence intensity of full length GFP-HP1γ (FL) in HEK293T cells before and after treatment with 3% 1,6-hexanediol (1,6-Hex) at different time. **b** Fluorescence recovery after photobleaching (FRAP) assay of HP1γ-GFP in HEK293T cells (mean ± s.d.; n= 3 independent experiments). **c** Western blotting shows the overexpression efficacy of HDAC1 in HEK293T cells. **d** Representative images for HP1γ foci in HEK293T cells with or without HDAC1 OE (mean ± s.d.; n= 3 independent experiments). Quantification of puncta count is shown in images. Two-sided P values were determined by Student's t test. **e** Analysis of the HP1γ protein sequence for intrinsically disordered regions using the program VL3-BA. Red dots, potential acetylated lysine sites. **f** Sequence alignment of CBX3 (HP1γ) -WT, K5Q, K5R and KallQ. The site of lysine

acetylation mutants indicated by red arrows. **g** Coomassie blue staining shows *in vitro* expression of GFP, (GFP-HP1γ) -WT, -K5Q, -K5R, -KallQ fusion protein, and **(h)** GST-GFP, (GST-GFP-HP1γ-IDR1) -WT, -K5Q, -K5R, -KallQ fusion protein. Source data are provided as a Source data file.

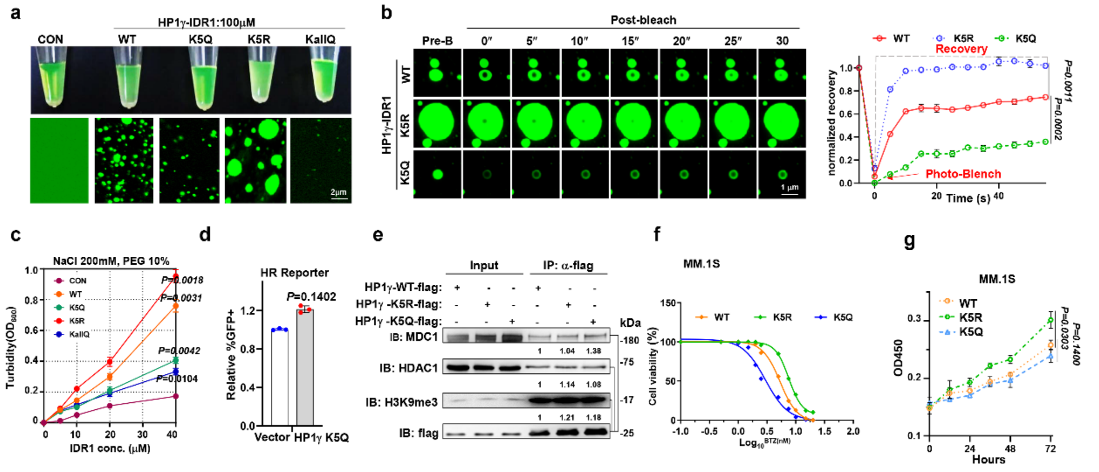

Supplementary Fig. 6: Enhancing nuclear condensation of HP1γ promote drug resistance to BTZ. a Visualization of turbidity associated with droplet formation *in vitro* for the GFP-vector control (Ctrl), (HP1γ-IDR1) -WT, K5Q, K5R or KallQ. **b** Microscopy images of GFP fusion proteins of (HP1γ-IDR1) -WT, -K5Q and -K5R before and after partial droplet photobleaching *in vitro* (mean ± s.d.; n= 3 independent experiments), and two-sided *P*-values of the comparisons between the final extent of recovery after photobleaching (i.e. 60 s) were performed using one-way ANOVA. **c** Turbidity (OD600) of WT, K5Q, K5R, and KallQ mutants of HP1γ-IDR1 in 200 mM NaCl and 10% PEG was measured (mean ± s.d.; n= 3 independent experiments). P values were determined by Pearson's coefficient and Student's *t* test. **d** Quantitative assessment of HR activity in the HEK293T cells expressing vector control or K5Q-HP1γ via flow cytometry assay for the percentage of GFP^+ cells among RFP^+ cells (mean ± s.d.; n= 3 independent experiments). **e** Co-IP assay for HP1γ-WT, -K5Q or -K5R with MDC1, HDAC1 and H3K9me3 in HEK293T cells. Input, 2% whole lysate; IP, M2-flag antibody. **f** Alteration of IC_{50} to BTZ treatment in the HP1γ-WT, -K5R and -K5Q in MM.1S cells (mean ± s.d.; n= 3 independent experiments). **g** Cell proliferation of MM.1S cells stably expressing HP1γ-WT, -K5Q or -K5R for 72 hr. *P* values were

determined by two-way ANOVA test **(c, g)** and Student's t test **(d)**. Source data are provided as a Source data file (mean ± s.d.; n= 3 independent experiments).

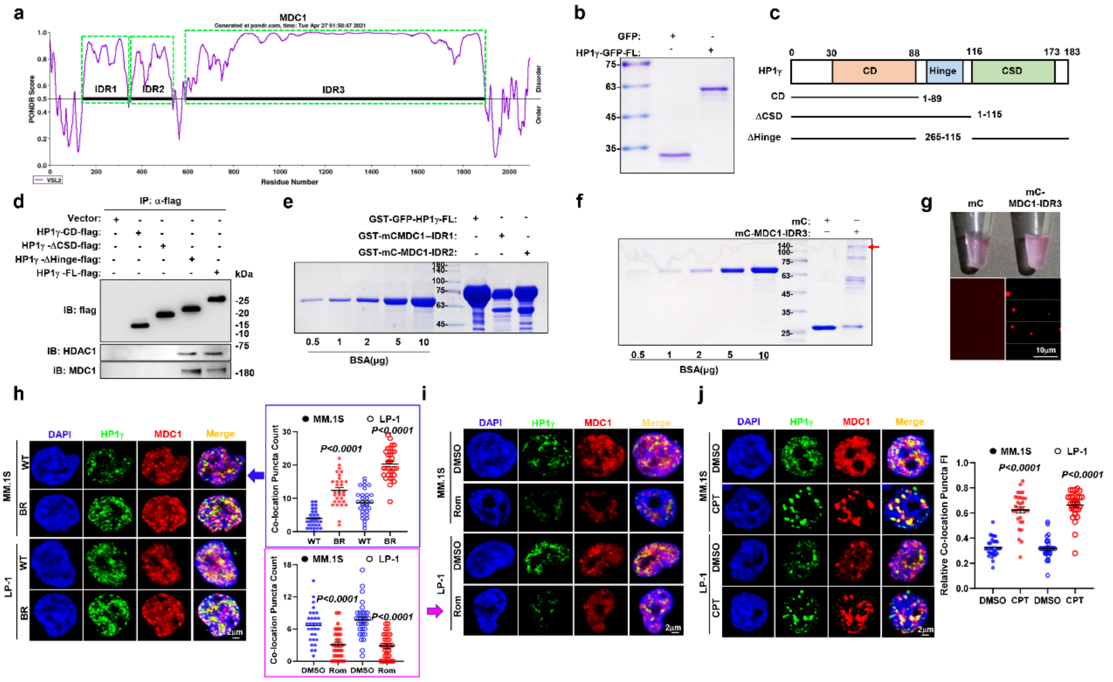

Supplementary Fig. 7: Interaction with MDC1-IDR1 enhances HP1γ droplet formation *in vitro*. **a** Analysis of the MDC1 protein sequence for intrinsically disordered regions using the program VSL2. **b** Coomassie blue staining to show in vitro expression of GFP and HP1γ-GFP-FL fusion protein. **c** Schematic illustration of strategy to construct HP1γ truncations. **d** Immunoprecipitation assay to show interactions of full length HP1γ (FL) or truncations with HDAC1 or MDC1 in HEK293T cells. **e** Coomassie blue staining to show *in vitro* expression of GST-GFP-HP1γ-FL, GST-MDC1-mC (mCherry) -IDR1 and GST-MDC1-mC-IDR1 protein, and **(f)** mC and mC-MDC1-IDR3 protein. **g** Visualization of turbidity associated with droplet formation *in vitro* for mC and mC-MDC1-IDR3 protein. **h** Representative images for HP1γ and MDC1 foci in WT and BR MM cells, and (**i**) MM cells treated with or without Rom, and (**j**) CPT. Quantification of puncta count or relative puncta FI (fluorescence intensities) in 30 cells are shown in images. Two-sided *P* calculated using Student's t test; mean ± s.d.; n= 3 independent experiments. Source data are provided as a Source data file.

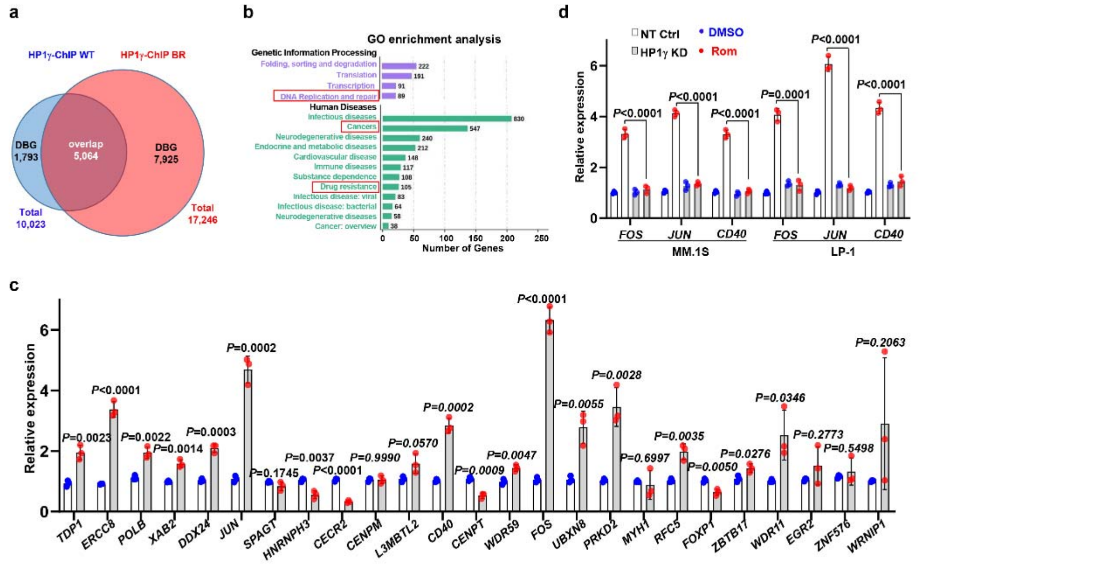

Supplementary Fig. 8: HP1 promotes transcription of resistance genes. a Venn diagram shows the number of overlapped genes between WT and BR cells in HPIγ-ChIP seq. **b** KEGG analysis for differentially expressed genes with a $P < 0.05$ using DAVID methods. **c** QPCR shows expression of selected genes which overlapped between ChIP seq and RNA seq in WT and BR MM cells, and (**d**) *FOS*, *JUN* and *CD40* genes expression in NT Ctrl and HP1γ KD cells treated with DMSO or Rom, mean ± s.d.; n= 3 independent experiments，and *P* values were determined by Student's t test. Source data are provided as a Source data file.

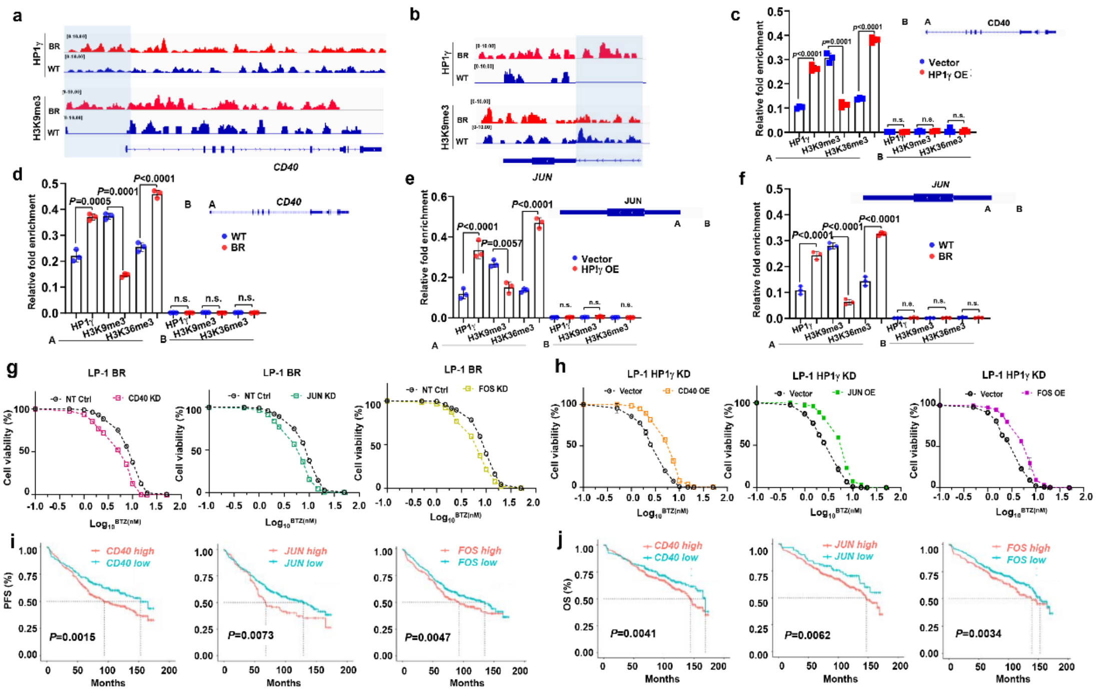

Supplementary Fig. 9: HP1γ facilitates chromatin accessibility of genes governing drug sensitivity. a Gene tracks showing representative ChIP-Seq profiles for the indicated proteins and histone marks at the *CD40*, and (**b**) *JUN* gene loci. ChIP-qPCR assay to show enrichment of HP1γ, H3K9me3 and H3K36me3 at gene loci of (**c**) *JUN,* and (**e**) *CD40* in the Vector and HP1γ overexpressing LP-1 cells, (**d**) *JUN,* and (**f**) *CD40* in WT and BR LP-1 cells (mean ± s.d.; n= 3 independent experiments). **g** Alteration of IC50 to BTZ treatment in the NT Ctrl, CD40 KD, JUN KD and FOS KD LP-1 cells (n = 3), and (**h**) in the Vector, CD40 OE, JUN OE and FOS OE LP-1 cells with HP1γ KD stably (mean ± s.d.; n= 3 independent experiments). **i** Correlation of *FOS, JUN* and *CD40* expression with progression free survival (PFS), (**j**) overall survival (OS) in patients after receiving BTZ-based treatment regimens from the coMMpass cohort. *P* values were determined by Pearson's coefficient and log-rank test. Source data are provided as a Source data file.

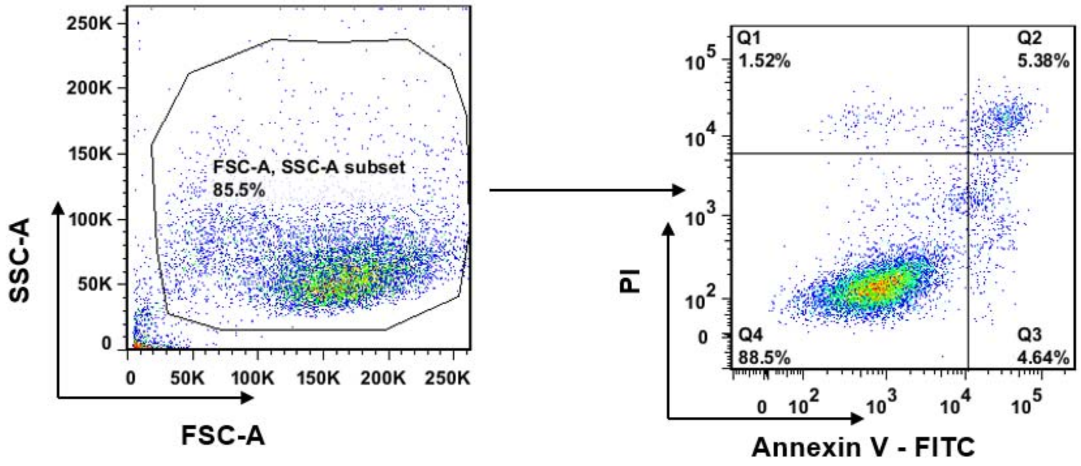

Supplementary Fig. 10: Flow cytometry gating schemes. a Gating scheme used for analysis of apoptosis. First debris were excluded (FSC-A vs SSC-A), and apoptosis cells were assessed for Annexin V and PI expression, then cells were separated into viability (Annexin V- PI-), early apoptosis (Annexin V+ PI-) and late apoptosis (Annexin V+ PI+). Gating is related to Fig. 1m, Fig. 8a and Supplementary Fig. 2d.

.

Supplementary Tables

Category	Source	Cat. No.
**Antibodies**		
Anti-rabbit HP1γ	Cell signaling technology	2619
Anti-rabbit Acetylated-Lysine	Cell signaling technology	9441
Anti-rabbit CBX3	Abclonal	A2248
Anti-mouse HP1γ,clone 42s2	millipore	05-690
Anti-rabbit GFP (D5.1)	Cell signaling technology	2956
Anti-rabbit HDAC1	Abclonal	A0238
Anti-rabbit HDAC2	Abclonal	A2084
Anti-mouse phospho-Histone H2A.X	millipore	05-636
Anti-mouse MDC1	millipore	05-1572
Anti-PARP	Cell signaling technology	9532
Anti-rabbit β-actin	Abclonal	AC006
Anti-mouse Ub	Cell signaling technology	3936
Anti-Histone H3 (tri methyl K9) antibody-ChIP Grade	Cell signaling technology	13969
Anti-H3K36me3 antibody [D5A7] - ChIP Grade	Cell signaling technology	4909
Anti-rabbit HA-Tag(C29F4)	Cell signaling technology	3724
Anti-rabbit GFP-Tag pAb	Abclonal	AE011
Anti-mouse GST-Tag mAb	Abclonal	AE001
Anti-rabbit KAP1/TRIM28 pAb	Abclonal	A2245
Anti-rabbit Phospho-p95/NBS1 (Ser343)	Cell signaling technology	3001
Anti-rabbit Phospho-ATM (Ser1981) (D6H9) mAb	Cell signaling technology	5883
Anti-rabbit GAPDH pAb	Abclonal	AC001
Anti-rabbit Histone H3 (D1H2) XP mAb	Cell signaling technology	4499
Goat Anti-Rabbit IgG-HRP	Sigma-Aldrich	A0545
ANTI-FLAG® M2-Peroxidase	Sigma-Aldrich	A8592
Rabbit Anti Mouse IgG-HRP	Sigma-Aldrich	A9044-2ML
Alexa Fluor® 555 donkey anti-rabbit IgG (H+L)	Invitrogen	A31572
Alexa Fluor® 488 donkey anti-rabbit IgG (H+L)	Invitrogen	A11034
Anti-rabbit IgG	Proteintech	30000-0-AP
Anti-mouse IgG	Proteintech	B900620
**Chemicals, Peptides and Recombinant Proteins**		
3FLAG peptide	Sigma-Aldrich	F4799
FLAG Peptide	Sigma-Aldrich	F3290

**Drugs**		
Bortezomib (PS-341)	SelleckChem	S1013
Romidepsin	MedChemExpress	HY-15149
Puromycin 2HCL	SelleckChem	S7417
Cycloheximide	Sigma-Aldrich	C7698
Camptothecin	Gift from Dr. Lei Shi, Tianjin Medical University	
**Enzymes**		
RNase A, DNase and protease-free	Thermo Fisher	EN0531
Proteinase K Solution, ChIP grade	Thermo Fisher	26160
Benzonase Nuclease	Sigma-Aldrich	E1014-25KU
NotI-HF	NewEngland Biolabs	R3189S
BamHI	NewEngland Biolabs	R0136S
EcoRI	NewEngland Biolabs	R0101S
KpnI-HF	NewEngland Biolabs	R3142S
XhoI	NewEngland Biolabs	R0146S
AgeI	NewEngland Biolabs	R3552S
CutSmart Buffer	NewEngland Biolabs	137204S
T4 DNA Ligase	NewEngland Biolabs	M0202S
10×Buffer for T4 DNA ligase	NewEngland Biolabs	B0202S
Multiscribe Reverse Transcriptase	ABI	4308228
dNTP mix	ABI	362275
**Plasmids**		
hU6-MSC-Ubiquitin-eGFP	Shanghai genechem	GV298
CBX3-shRNA1	Shanghai genechem	PSC57295-1
CBX3-shRNA2	Shanghai genechem	PSC57297-1
CBX3-shRNA3	Shanghai genechem	PSC57296-1
pLKO.1		
HDAC1-flag	Gift from Dr. Dr. Robert Orlowski, UT MD Anderson Cancer Center	
HR-GFP receptor	Gift from Dr. Jun Li, Peking Union Medical College	
NHEJ-GFP receptor	Gift from Dr. Jun Li, Peking Union Medical College	
pcDNA-3×FALG	Gift from Dr. Michael Naksi lab, UT Health Science Center at San Antonio	
pITA	Gift from Dr. Yupeng Chen, Tianjin Medical University	
$psPAX_2$	Gift from Dr. Xudong Wu, Tianjin Medical University, Dept. Cell Biology	
pMD2.G	Gift from Dr. Xudong Wu, Tianjin Medical University, Dept. Cell Biology	
pEGFP-C1	Gift from Dr. Yupeng Chen, Tianjin Medical University	
pGEX-6p-1-GFP-A206K	Gift from Dr. Yupeng Chen, Tianjin Medical University	
pITA insert-CBX3-N-FLAG	Self-construction	
pITA insert-CBX3-C-FLAG	Self-construction	
pEGFP-C1-CBX3-WT	Self-construction	

pEGFP-C1-CBX3-K5Q	Self-construction	
pEGFP-C1-CBX3-K5R	Self-construction	
pGEX-5X-3-CBX3	Self-construction	
pGEX-6p-1-GFP-A206K-CBX3-IDR1-WT	Self-construction	
pGEX-6p-1-GFP-A206K-CBX3-IDR1-K5Q	Self-construction	
pGEX-6p-1-GFP-A206K-CBX3-IDR1-K5R	Self-construction	
pGEX-6p-1-GFP-A206K-CBX3-IDR1-KAllQ	Self-construction	
MDC1 shRNA1	Self-construction	
MDC1 shRNA2	Self-construction	
**Critical Commercial Assays**		
EvaGreen 2X qPCR MasterMix	ABI	MasterMix-R
5×All-In-One RT MasterMix	abm	G490
Pierce BCA Protein Assay Kit	Thermo SCIENTIFIC	23225
AxyPrep DNA Extraction Kit	AXYGEN	295 AP-GX-250G
AxyPrep Plasmid Miniprep Kit	AXYGEN	183 AP-MN-P-250G
Plasmid Maxi Kit (25)	QIAGEN	12163
EnVision G12 Doublestain System,Rabbit/Mouse(DAB+/Permanent Red)	Dako	K5361
SuperSignal West Dura Extended Duration Substrate	ThermoFisher	34580
9002 SimpleCHIP® Kit	Cell Signaling	22188S
Simple CHIP® Kits-20C-Reagents	Cell Signaling	45061S
ChIP-grade Protein A/G Magnetic Beads	Thermo SCIENTIFIC	26162
ANTI-FLAG M2 Affinity Gel	Sigma-Aldrich	A2220
CellTiter 96 Aqueous One Solution	Promega	G358B
NuPAGE 4-12% Bis-Tris Gel	Invitrogen	NP0335BOX
Phosphatase Inhibitor Cocktail (100×)	Cell Signaling	587OS
Annexin V-FITC Apoptosis Kit	Sigma-Aldrich	APOAF-50TST
Pierce® Protein G Plus Agarose	Thermo Scientific	22852
Human CD20 MicroBeads	Miltenyi Biotec	MB17-R0829
Human CD138 MicroBeads	Miltenyi Biotec	130-105-961
DeadEnd™ Fluorometric TUNEL System	Promaga	G3250
Ficoll-Paque PLUS endotoxin tested	GE Healthcare	17-1440-02
LS Columns (25 columns)	Miltenyi Biotec	130-042-401
TRIzol Reagent	Ambion, Life Science	15596018
Opti-MEM®I (1×) Reduced Serum	Gibco, Life Technologies	31985-070

Cell Cycle and Apoptosis Analysis Kit	Beyotime	C1052
Opti-protein XL Marker	ABM	G266
PageRuler Prestained protein Ladder	ThermoFisher Scientific	26616
1Kb Ladder DNA Marker	Biomed	MD114
1Kb DNA Ladder	TIANCEN	MD111
100bp DNA Ladder	TRANS	BM301
BM15000 DNA Marker	Biomed	MD106
1Kb Plus DNA Ladder	Solarbio	M1500
PEI-Transferrinfection Kit	ThermoFisher Scientific	BMS1003
**Primer sequences**		
Human GAPDH-F	TTGCCCTCAACGACCACTTT	
Human GAPDH-R	TGGTCCAGGGGTCTTACTCC	
Human CBX3-F	TGCTGCTGACAAACCAAGAG	
Human CBX3-R	CACCAAGTCTGCCTCATCTG	
Human CBX5-F	GGCAACAGATTCCTGTGGTG	
Human CBX5-R	GCATGCCATGTCAGTCTCTC	
Human CBX1-F	AAGGGAGAGGAGAGCAAACC	
Human CBX1-R	TTGACATTGGCTTCCTTGGC	
Mouse Fos-F	TGTGTTCCTGGCAATAGCGT	
Mouse Fos-R	ACCACCTCGACAATGCATGA	
Mouse Jun-F	GGGAGCATTTGGAGAGTCCC	
Mouse Jun-R	TTTGCAAAAGTTCGCTCCCG	
Mouse Cd40-F	TTGTTGACAGCGGTCCATCT	
Mouse Cd40-R	CACATGCCTCGCAATCCTTG	
Human FOS-F	GGGGCAAGGTGGAACAGTTAT	
Human FOS-R	AGGTTGGCAATCTCGGTCTG	
Human JUN-F	GCCAGGTCGGCAGTATAGTC	
Human JUN-R	GGACTCTGCCACTTGTCTCC	
Human CD40-F	ACTGATGTTGTCTGTGGTCCC	
Human CD40-R	GGCCACCTTTTTGATAAAGACCAGC	
FOS-A-F	GCGGCCGGGCCGGGGAGGCC	
FOS-A-R	CCCTTCTCCACTCTTCGGGC	
FOS-B-F	ACACCACTGCACTCCAGCCT	
FOS-B-R	GCCTACGTAAATGCTGCCGC	
JUN-A-F	GCAACTCGGCGCGGATGGAG	
JUN-A-R	GGGGCAGTTTTTATAGTGGG	
JUN-B-F	GCAACTCGGCGCGGATGGAG	
JUN-B-R	GGGCAGTTTTTATAGTGGGC	
CD40-A-F	TTCCATCCTCCCTGATCTGTGAGGT	
CD40-A-R	CTCCACGTTGGCCAGGCTGGTCTCA	
CD40-B-F	GGGGAGAAAATCTTCATCAT	
CD40-B-R	CTTTCTCACCCTTCAAGGTC	
pITA-CBX3-N-flag-KpnI-F	CGGGGTACCATGGATTACAAGGATGACGACGA	

	TAAGGCCTCCAACAAAACTAC
pITA-CBX3-N-flag-BamHI-R	CGCGGATCCTTATTGAGCTTCATCTTCTG
pITA-CBX3-C-flag-KpnI-F	CGGGGTACCATGGCCTCCAACAAAACTACATT
pITA-CBX3-C-flag-BamHI-R	CGCGGATCCTTACTTATCGTCGTCATCCTTGTA ATCTTGAGCTTCATCTTCTGG
pEGFP-C1-CBX3-C-flag-KpnI-F	CGGGGTACCATGGCCTCCAACAAAACTACATT
pEGFP-C1-CBX3-C-flag-BamHI-R	CGCGGATCCTTACTTATCGTCGTCATCCTTGTA ATCTTGAGCTTCATCTTCTGG
pEGFP-C1-CBX3-K5Q-F	CGATAAGGCCTCCAACCAGACTACA
pEGFP-C1-CBX3-K5Q-R	TTTTTTGCAATGTAGTCTGGTTGGA
pEGFP-C1-CBX3-K5R-F	CGATAAGGCCTCCAACAGGACTACA
pEGFP-C1-CBX3-K5R-R	TTTTTTGCAATGTAGTCCTGTTGGA
pGEX-5X-3-CBX3-BamHI-F	GCGGATCCATGGCCTCCAACAAAACTACAT
pGEX-5X-3-CBX3-XhoI-R	CCGCTCGAGTTATTGAGCTTCATCTTCTGG
pGEX-6p-1-GFP-A206K-CBX3-IDR1-WT-XhoI-F	CCGCTCGAGATGGCCTCCAACAAAAC
pGEX-6p-1-GFP-A206K-CBX3-IDR1-WT-NotI-R	ATAAGAATGCGGCCGCTTAATTCACTACACGT CGATC
pGEX-6p-1-GFP-A206K-CBX3-IDR1-K5Q-XhoI-F	CCGCTCGAGATGGCCTCCAACCAGAC
pGEX-6p-1-GFP-A206K-CBX3-IDR1-K5Q-NotI-R	ATAAGAATGCGGCCGCTTAATTCACTACACGT CGATC
pGEX-6p-1-GFP-A206K-CBX3-IDR1-K5R-XhoI-F	CCGCTCGAGATGGCCTCCAACAGGAC
pGEX-6p-1-GFP-A206K-CBX3-IDR1-K5R-NotI-R	ATAAGAATGCGGCCGCTTAATTCACTACACGT CGATC
pGEX-6p-1-GFP-A206K-CBX3-IDR1-KAllQ-XhoI-F	CCGCTCGAGGCCTCCAACCAAACTACATTGCA ACAAATGGGACAACAACAGAATGGACAGAGT CAACAA
pGEX-6p-1-GFP-A206K-CBX3-IDR1-KAllQ-M	CAGAGTCAACAAGTTGAAGAGGCAGAGCCTGA AGAATTTGTCGTGGAACAAGTACTAGAT
pGEX-6p-1-GFP-A206K-CBX3-IDR1-KAllQ-NotI-R	ATTTGCGGCCGCCTATTAATTCACTACACGTCG ATCTAGTACCTG
Human MDC1-shRNA1-F	CCGGCCCTGAATCAACTGTCCCTATCTCGAGAT AGGGACAGTTGATTCAGGGTTTTTG
Human MDC1-shRNA1-R	AATTCAAAAACCCTGAATCAACTGTCCCTATCT CGAGATAGGGACAGTTGATTCAGGG
Human MDC1-shRNA3-F	CCGGCCTCAGTAGTTGCAGATGTAACTCGAGTT ACATCTGCAACTACTGAGGTTTTTG
Human MDC1-shRNA3-R	AATTCAAAAACCTCAGTAGTTGCAGATGTAAC TCGAGTTACATCTGCAACTACTGAGG
