# Supplementary material for: Deacetylation induced nuclear condensation of HP1γ promotes multiple myeloma drug resistance
Source: Nat Commun. 2023 Mar 9;14:1290. doi: 10.1038/s41467-023-37013-x (PMC9998874; doi:10.1038/s41467-023-37013-x)
Supplement: Supplementary file 3 — Reporting Summary [file 41467_2023_37013_MOESM3_ESM.docx]

1.) Please fill out the legends for the attached Description of Additional document.

**Response:** As requested, the description of the additional document has been attached.

2.) Please denote the Corresponding Author with a symbol next to the name and repeated prior to contact details

**Response:** We added a symbol to the corresponding author and repeated in the contact details.

3.) In the data availability section, please add also the accession codes of datasets already available that you used to generate new data as well (example: Multiple Myeloma 89 Research Foundation (MMRF), 542 MM patients (GSE9782)) in the line of “The XX publicly available data used in this study are available in the YY database under accession code ZZ [Add hyperlink here] [citation].”

**Response:** We followed your suggestion and added this description in the data availability section.

4.) For the accession code provided please add its hyperlink throughout (for example, "5XRN [http://doi.org/10.2210/pdb5XRN/pdb]", "1483958 [https://doi.org/10.5517/ccdc.csd.cc1lt5m6]", "SRP109982 [https://www.ncbi.nlm.nih.gov/sra/?term=SRP109982]", "GSE101099 [https://www.ncbi.nlm.nih.gov/geo/query/acc.cgi?acc=GSE10 1099]" or "NQLW00000000 [https://www.ncbi.nlm.nih.gov/assembly/GCA_002312845.1/]" ).

**Response:** We checked our accession codes and added their hyperlinks.

5.) Proteomics data must be deposited in PRIDE. If there are any restrictions, please add in the Data availability section, explanation of the nature of and reasons for the restrictions, and details of the conditions under which the data can be accessed or reused.

**Response:** We explained the reason for the restriction in the data availability section.

**Data availability**

The RNA-seq and ATAC-seq data can be publicly found at the Gene Expression Omnibus database under accession number [GSE176547](https://www.ncbi.nlm.nih.gov/geo/query/acc.cgi?acc=GSE176547) [https://www.ncbi.nlm.nih.gov/geo/query/acc.cgi?acc=GSE176547]. The Multiple Myeloma Research Foundation publicly available data used in this study are available in the Gene Expression Omnibus database under accession code [GSE2658](https://www.ncbi.nlm.nih.gov/geo/query/acc.cgi) [https://www.ncbi.nlm.nih.gov/geo/query/acc.cgi] and [GSE9782](https://www.ncbi.nlm.nih.gov/geo/query/acc.cgi) [https://www.ncbi.nlm.nih.gov/geo/query/acc.cgi]. The source data of proteomics can be available and shared after the year of 2023 due to an ongoing project correlated with this data. For data accession, please contact the corresponding author under a material transfer agreement with Tianjin Medical University. Source data are provided with this paper. Requests for any materials in this study should be directed to Zhiqiang Liu and obtained through an MTA.
