# Supplementary material for: Deacetylation induced nuclear condensation of HP1γ promotes multiple myeloma drug resistance
Source: Nat Commun. 2023 Mar 9;14:1290. doi: 10.1038/s41467-023-37013-x (PMC9998874; doi:10.1038/s41467-023-37013-x)
Supplement: Supplementary file 4 — Description of Additional Supplementary Files [file 41467_2023_37013_MOESM4_ESM.docx]

**Description of Additional Supplementary Files**

Supplementary Data 1

Description: Differentially expressed genes from transcriptome of the CON (Vector) and OE CBX3 (HP1γ OE) LP-1 cells

Supplementary Data 2

Description: List of overlapped genes in HP1γ-ChIP sequencing and RNA sequencing of HP1γ-overexpressing LP-1cells, and the gene ontology analysis of those gene.

Supplementary Data 3

Description: List of protein profile of the mass spectrum analysis of HP1γ-flag-pull down in the WT and BR LP-1 cells.
